# Neuroanatomical correlates of screening for aphasia in NeuroDegeneration (SAND) battery in non-fluent/agrammatic variant of primary progressive aphasia

**DOI:** 10.3389/fnagi.2022.942095

**Published:** 2022-10-31

**Authors:** Enrico Premi, Maria Cotelli, Elena Gobbi, Ilaria Pagnoni, Giuliano Binetti, Yasmine Gadola, Ilenia Libri, Irene Mattioli, Marta Pengo, Armin Iraji, Vince D. Calhoun, Antonella Alberici, Barbara Borroni, Rosa Manenti

**Affiliations:** ^1^Stroke Unit, Azienda Socio Sanitaria Territoriale Spedali Civili Brescia, Brescia, Italy; ^2^Neuropsychology Unit, IRCCS Istituto Centro San Giovanni di Dio Fatebenefratelli, Brescia, Italy; ^3^MAC Memory Clinic and Molecular Markers Laboratory, IRCCS Istituto Centro San Giovanni di Dio Fatebenefratelli, Brescia, Italy; ^4^Neurology Unit, Department of Clinical and Experimental Sciences, University of Brescia, Owensboro, Italy; ^5^Tri-Institutional Center for Translational Research in Neuroimaging and Data Science (TReNDS), Georgia Institute of Technology, Georgia State University, Emory University, Atlanta, GA, United States; ^6^Departments of Psychology and Computer Science, Georgia State University, Atlanta, GA, United States; ^7^Department of Electrical and Computer Engineering, Georgia Institute of Technology, Atlanta, GA, United States

**Keywords:** frontotemporal lobar degeneration, language, imaging, primary progressive aphasia, source-based morphometry (SBM), voxel-based morphometry (VBM)

## Abstract

**Background:**

Non-fluent/agrammatic variant of Primary Progressive Aphasia (avPPA) is primarily characterized by language impairment due to atrophy of the inferior frontal gyrus and the insula cortex in the dominant hemisphere. The Screening for Aphasia in NeuroDegeneration (SAND) battery has been recently proposed as a screening tool for PPA, with several tasks designed to be specific for different language features. Applying multivariate approaches to neuroimaging data and verbal fluency tasks, Aachener Aphasie Test (AAT) naming subtest and SAND data may help in elucidating the neuroanatomical correlates of language deficits in avPPA.

**Objective:**

To investigate the neuroanatomical correlates of language deficits in avPPA using verbal fluency tasks, AAT naming subtest and SAND scores as proxies of brain structural imaging abnormalities.

**Methods:**

Thirty-one avPPA patients were consecutively enrolled and underwent extensive neuropsychological assessment and MRI scan. Raw scores of verbal fluency tasks, AAT naming subtest, and SAND subtests, namely living and non-living picture naming, auditory sentence comprehension, single-word comprehension, words and non-words repetition and sentence repetition, were used as proxies to explore structural (gray matter volume) neuroanatomical correlates. We assessed univariate (voxel-based morphometry, VBM) as well as multivariate (source-based morphometry, SBM) approaches. Age, gender, educational level, and disease severity were considered nuisance variables.

**Results:**

SAND picture naming (total, living and non-living scores) and AAT naming scores showed a direct correlation with the left temporal network derived from SBM. At univariate analysis, the left middle temporal gyrus was directly correlated with SAND picture naming (total and non-living scores) and AAT naming score. When words and non-words repetition (total score) was considered, a direct correlation with the left temporal network (SBM) and with the left fusiform gyrus (VBM) was also evident.

**Conclusion:**

Naming impairments that characterize avPPA are related to specific network-based involvement of the left temporal network, potentially expanding our knowledge on the neuroanatomical basis of this neurodegenerative condition.

## Introduction

Primary Progressive Aphasia (PPA) is a neurodegenerative syndrome characterized by insidiously, predominant and progressive language impairment for at least two years after symptom onset, and it involves specific areas and networks of the left brain hemisphere (Bonner et al., 2010; Gorno-Tempini et al., 2011; Montembeault et al., 2018). Among the variants of PPA identified by the international guidelines, the non-fluent/agrammatic variant (avPPA), a clinical entity of the frontotemporal lobar degeneration (FTLD) spectrum, is described. In particular, it is characterized by slow, effortful and hesitant language production. Agrammatism and speech sound errors with distortions (apraxia of speech, AOS) are the core criteria of this variant and at least one of them should be present for the diagnosis (Gorno-Tempini et al., 2011). Typically, agrammatism consists of short sentences, in which the omission of grammatical morphemes is evident (Rohrer et al., 2010; Marshall et al., 2018). Speech sound errors refer to phonetic errors (i.e., distortions caused by AOS) or to phonemic errors (i.e., deletions, substitutions, insertions, and transpositions caused by phoneme selection deficit) in speech production (Ogar et al., 2007; Ash et al., 2013). Specifically, these types of errors are frequently observed during confrontation naming tasks, resulting from a difficulty in the phoneme selection process or/and phonetic errors caused by progressive articulation planning deficit (Jordan and Hillis, 2006; Ash et al., 2010; Budd et al., 2010). Consequently, difficulty in naming is a feature commonly found in avPPA patients, reflecting damage to the postlexical level of word production (Ash et al., 2010; Mack et al., 2013).

In addition, according to the current clinical criteria for diagnosis of the avPPA variant, at least two clinical features among impairment of grammatically complex sentence comprehension, preservation of single-word comprehension and spared object knowledge must be present (Gorno-Tempini et al., 2011). At the neuroanatomical level, this variant appears to be characterized by involvement of the left frontal lobe extending to the insula and anterior superior temporal regions along with the damage of the white-matter dorsal language pathway connecting the frontal, subcortical and parietal regions as well as a damage in the superior longitudinal fasciculus (Rohrer et al., 2010; Galantucci et al., 2011; Gorno-Tempini et al., 2011; Grossman, 2012; Agosta et al., 2013; Mandelli et al., 2014; Marshall et al., 2018; Montembeault et al., 2018).

To date, there is high heterogeneity in the selection and administration of language tests used for PPAs diagnosis (Henry and Grasso, 2018; Tippett, 2020). In clinical practice, most of the language tests used have been developed primarily for the assessment of aphasia due to stroke and often each test examines one language domain. As a result, different language scores are usually combined to help the diagnosis, leading often to difficult interpretations due to differences in normative groups and task properties across tests. Moreover, these tests may sometimes lack sensitivity to detect the specific deficits present in PPA (Savage et al., 2013). To overcome this problem, recently, some authors developed new batteries, with the aim of harmonizing the diagnostic process between different centers (Savage et al., 2013; Catricalà et al., 2017; Epelbaum et al., 2020; Patel et al., 2022). In particular, Catricalà et al. (2017) implemented a new screening battery, the Screening of Aphasia for NeuroDegneration (SAND), capable of capturing the key language features required for the diagnosis and classification of PPAs through the assessment of different components of language. The battery incorporates a set of tests adapted to measure specific linguistic domains in PPA, including assessments of lexical retrieval, syntax and semantic processes. Moreover, it proposes a performance classification based on the quantitative and qualitative error analysis to better identify the nature of the language dysfunctions related to a specific variant of PPA (Catricalà et al., 2017; Battista et al., 2018; Patel et al., 2022). Since this brief battery was developed taking into account the psycholinguistic factors that can affect PPA patients' performance, it might represent a valuable screening tool for detecting language impairment in neurodegenerative disorders (Catricalà et al., 2017; Battista et al., 2018). Moreover, the development of language measures designed considering the specific language features of neurodegenerative disorders could also represent a great opportunity to better shed light on the brain correlates in the PPA (Brambati et al., 2006; Amici et al., 2007; Breining et al., 2022). In this regard, univariate neuroimaging approach like voxel-based morphometry (VBM) (Ashburner and Friston, 2000; Good et al., 2001) has been used in past to explore the neuroanatomical underpinnings of language functioning in neurodegenerative diseases (Brambati et al., 2006; Amici et al., 2007; Gunawardena et al., 2010; Rogalski et al., 2011; Breining et al., 2022). In recent years, a multivariate approach (named source-based morphometry, SBM) (Xu et al., 2009; Gupta et al., 2019) based on independent component analysis (ICA) has been applied to structural data to obtain non-overlapping spatial components. These covariance networks represent brain regions that are working together, showing a greater structural affinity based on mutually trophic effects or shared mechanisms of experience-related plasticity (He et al., 2009; Montembeault et al., 2012; Alexander-Bloch et al., 2013; Evans, 2013; Fornito et al., 2015). Due to its data-driven nature, ICA can help in removing artifacts from real data, potentially increasing the signal-to-noise ratio (Zeman et al., 2007; Chen et al., 2014).

Taking into account these assumptions, the aim of the present study is to evaluate the univariate (VBM) and multivariate (SBM) structural correlation of the linguistic scores recorded using verbal fluency tasks, AAT naming subtest, and SAND subtests in a group of avPPA patients to elucidate the neuroanatomical correlates of language deficits in avPPA. For the correlation analysis, we used tasks highly recommended for avPPA patients, which are frequently able to detect difficulties in these subjects (picture naming tests, auditory sentence comprehension tests, words and non-words repetition, sentence repetition tests and verbal fluency tasks). Moreover, a task generally spared in avPPA like single-word comprehension was also considered as internal control potentially increasing the specificity of the correlation analysis (Gorno-Tempini et al., 2011; Montembeault et al., 2018).

## Methods

### Participants

Thirty-one patients who had previously been diagnosed with avPPA by experienced neurologists were included in the study. Differential diagnosis was conducted by the neurologists based on clinical, neuropsychological and language examinations and all patients fulfilled the diagnostic criteria for imaging-supported avPPA (left posterior fronto-insular atrophy or hypometabolism) (Gorno-Tempini et al., 2011). The participants were recruited through the main clinical trial (ClinicalTrials.gov identifier: NCT04187391) from MAC Memory Clinic at IRCCS Istituto Centro San Giovanni di Dio, Fatebenefratelli and from the Center for Neurodegenerative Disorders, Department of Clinical and Experimental Sciences, University of Brescia, Italy. All participants were native Italian speakers and had normal or corrected-to-normal visual acuity. Moreover, all were right-handed, except for a subject who was ambidextrous (Table 1). All patients included had no other neurological (e.g., cerebrovascular disorders, previous stroke, hydrocephalus, and intracranial mass) or psychiatric disorders.

**Table 1:** Demographical and clinical features of the avPPA sample.

	**avPPA**
	**(*n* = 31)**
	**Mean (SD)**
Age (years)	68.2 (8.2)
Education (years)	11.0 (4.6)
Gender (males/females)	11/20
Symptom duration (months)	30.5 (19.7)
Edinburgh Handedness Inventory (%)	83.6 (24.9)

Mean and standard deviation (SD) between brackets are reported.

The study had ethical approval from the local Human Ethics Committee and was conducted in accordance with the Declaration of Helsinki. Before being enrolled in the study, all participants were informed about the aim of the study and signed written informed consent.

### Clinical, neuropsychological and language assessment

#### Clinical assessment

Family history of dementia, medical events, current medication and complete neurologic examination results were recorded, and the Cognitive Reserve Index questionnaire (CRIq) (Nucci et al., 2012) and the FTLD-modified Clinical Dementia Rating scale (FTLD-modified CDR) (Knopman et al., 2008; Borroni et al., 2010) were completed.

At the assessment visit, the evaluation of communication and functional abilities is conducted using the Stroke and Aphasia Quality of Life Scale (SAQOL-39) (Hilari et al., 2003). The SAQOL-39 is based on four subdomains: physical, psychosocial/mood, communication, and energy. The score in each subdomain ranges from 0 (higher difficulties) to 5 (no difficulties). The Speech Questionnaire (Lincoln, 1982) and Communication Assessment Scale according to Goodglass and Kaplan were also applied (Posteraro et al., 2006). Depression was assessed with the Beck Depression Inventory (BDI-II) (Beck et al., 1996), whereas personality and behavior changes were recorded using the Frontal Behavioral Inventory (FBI) (Alberici et al., 2007).

#### Neuropsychological assessment

The neuropsychological assessment included the Mini-Mental State Examination (MMSE) (Folstein et al., 1975) for the assessment of global cognition; the Story Recall (Novelli et al., 1986) and the Rey-Osterrieth complex figure test-recall (Caffarra et al., 2002) for episodic memory; phonemic and semantic verbal fluency (Novelli et al., 1986) for language production; the Rey-Osterrieth complex figure test-copy (Caffarra et al., 2002) for visuo-constructional abilities and the Trail Making Test (TMT) part A and part B (Giovagnoli et al., 1996; Siciliano et al., 2019) for attention functions.

All the tests were administered and scored according to standard procedures (Lezak et al., 2004).

#### Language assessment

Linguistic abilities were evaluated through the naming subtest of the Aachener Aphasie Test (AAT) (Luzzatti et al., 1994), an object and action naming task (International Picture Naming Project, IPNP, Bates et al., 2000) and the Screening for Aphasia in NeuroDegeneration battery (SAND) (Catricalà et al., 2017). The SAND is a screening battery for language assessment, capable of capturing the key language features required for the diagnosis and classification of PPA and it includes nine tests: picture naming, auditory sentence comprehension, single-word comprehension, words and non-words repetition, sentence repetition, reading, writing, semantic association and picture description.

In the *picture naming test*, the subject is requested to name 14 black and white drawings (*total score range:* 0–14), each presented for 6 seconds. The pictures include seven living and seven non-living items, allowing a distinction between the two categories during the correction procedure (*living score range*: 0–7; *non-living score range:* 0–7). One point is given for each correct response, 0.5 points when the correct response is given after the phonological cue; 0 for incorrect responses or failure to respond (quantitative analysis). In addition to the quantitative assessment of task performance, it is possible to report the type of errors committed (distortions and phonological, visual and semantic errors) to allow a qualitative assessment of performance.

The *auditory sentence comprehension test* includes eight sentences, with four different syntactic complexity structures (short active, short passive, coordinate and embedded sentences; *total score range:* 0–8). The subject is asked to select which picture (between the target picture and the morphological/thematic distractor) corresponds to the sentence read by the examiner. One point is given for a correct answer and 0 for an incorrect answer (quantitative analysis). A qualitative evaluation of the performance can be made by distinguishing errors in selecting the morphological distractor from errors in choosing the thematic distractor.

In the *single-word comprehension test*, four pictures are presented, one target and three distractors and the participant is asked to point to the figure that represents the word spoken by the examiner. The test includes twelve trials (*total score range:* 0–12), six involving living and six involving non-living pictures, allowing independent scores for the living and non-living categories (*living score range*: 0–6; *non-living score range:* 0–6). One point is given for the correct answer, 0 for an incorrect answer (quantitative analysis).

The *words and non-words repetition test* consist of 10 items (*total score range: 0–10):* six words *(range score: 0–6)* and four non-words *(range score: 0–4)*. The subject is requested to repeat the item read by the examiner. One point is given for the correct answer and 0 for an incorrect answer (quantitative analysis). For this test, a qualitative assessment of task performance could also be carried out, reporting the presence of distortions and phonological, morphological, semantic and lexicalization errors, as well as omissions.

Likewise, in the *sentence repetition test*, six sentences are read to the patient *(total score range: 0–6)*, three predictable *(range score: 0–3)* and three unpredictable *(range score: 0–3)*, asking him to repeat the sentences read by the examiner. One point is given for the correct answer, 0 for an incorrect answer (quantitative analysis). Further to the quantitative assessment, it is recommended to report the type of errors made (distortions and phonological, morphological and semantic errors) as well as omissions to allow a qualitative analysis.

In the *reading test*, the subject is requested to read sixteen items *(total score range: 0–16)*, of which twelve words *(range score: 0–12)* and four non-words *(range score: 0–4)*. One point is given for the correct answer, 0 for an incorrect answer (quantitative analysis). In addition to the quantitative assessment, also for this test, it is possible to carry out a qualitative assessment of task performance, reporting the presence of distortions and phonological, semantic and morphological errors, regularizations, lexicalization errors and omissions.

For the *writing test*, the subject is asked to describe how to brush their teeth. For the quantitative analysis of this task, two different scores are possible. In detail, for fast scoring, the number of produced Information Units (*IU range score: 0–6)* is considered. One point is given for each Information Unit correctly identified. For a more detailed and optional scoring, six language features are analyzed (number of words number of nouns/number of total words; number of verbs/number of total words; number of correct syntactic structures/total number of syntactic structures; number of orthographic errors; lexical-semantic errors/number of words). In addition, the presence of modifications or errors (allography, micrography, orthographic, semantic, and grammatical/ syntactic errors) can also be reported for a qualitative analysis of the writing production.

The *semantic association test* includes four trials, each involving three images, and the patient is asked to point at the two semantically related *(total score range: 0–4)*. One point is given for the correct answer, and 0 for an incorrect answer.

In the *picture description test*, an individual is requested to describe a picture of a seaside scene. As writing, for the assessment of test performance, it is possible to a faster quantitative scoring, counting the number of produced Information Units *(IU range score*: 0–8), in which one point is given for each correctly identified Information Unit and an optional scoring that includes eight language features (number of words; number of nouns/number of total words; number of verbs/number of total words; total number of syntactic structures; number of subordinates/total number of syntactic structures; number of repaired sequences/number of words; number of phonological errors/number of words; lexical-semantic errors/number of words). Moreover, the presence of modifications or errors (articulatory, phonological, semantic, and grammatical/syntactic errors, hesitations/false starts/repaired sequences) must be reported for a qualitative analysis of the performance.

The entire battery takes < 20 min to administer.

### Structural magnetic resonance imaging (MRI)

#### Acquisition and pre-processing

Brain structural images (three-dimensional T1-weighted Magnetization Prepared—RApid Gradient Echo (MPRAGE) MRI) were collected using a 3-tesla Siemens Skyra scanner. As the first step, the raw DICOM scans were converted into the Neuroimaging Informatics Technology Initiative format, using MRIcroGL software (www.nitrc.org/projects/mricrogl). T1-weighted images were then processed and analyzed with the voxel-based morphometry (VBM) pipeline implemented in the Computational Anatomy Toolbox (CAT12 v.1742) (www.neuro.uni-jena.de/cat) for Statistical Parametric Mapping (SPM12, v.7219) (www.fil.ion.ucl.ac.uk/spm/software/spm12) running on MATLAB R2019b (the MathWorks, Inc., Natick, Massachusetts, United States). The VBM pipeline consists of several stages (tissue segmentation, spatial normalization to a standard Montreal National Institute [MNI] template, modulation, and smoothing), as previously described (Kurth et al., 2015). CAT12 potentially provided more robust and accurate performances compared to other VBM pipelines (Farokhian et al., 2017) in the calculation of gray matter volume (GMV). The normalized and modulated gray matter images were then smoothed with 10-mm full width at half-maximum Gaussian kernel.

#### Univariate analysis (VBM with SPM)

A multiple regression model was implemented in SPM for each selected test. Age, gender, educational level (years of schooling), total GMV and FTLD-modified CDR (sum of boxes) were entered as nuisance variables in the model. A statistical threshold of *p* < 0.05 corrected for multiple comparisons (whole-brain FWE) was adopted.

#### Source based morphometry (SBM)

SBM was initially described to study co-varying patterns of alterations in MRI (i.e., gray matter density Xu et al., 2009), cortical thickness (Steenwijk et al., 2016), fractional anisotropy (Caprihan et al., 2011) in different conditions like healthy aging (Eckert et al., 2010), schizophrenia (Xu et al., 2009; Gupta et al., 2015) and Parkinson's Disease (Rektorova et al., 2014; Premi et al., 2017). SBM leverages independent component analysis (ICA) to extract spatially independent patterns that occur in structural images. In contrast to mass-univariate testing (i.e., VBM analysis), SBM captures interrelationships between voxels to identify patterns of structural variation. Furthermore, as a multivariate approach, SBM can result in less-noisy sources of interest as well as a reduced number of multiple comparisons (Xu et al., 2009). Pre-processed GMV images (normalized, modulated, and smoothed) were considered for SBM analysis. Briefly, SBM used spatial independent component analysis (ICA) to decompose GMV variation across subjects into sources of common variance, considering a subjects-by-voxels data matrix. In line with the original paper (Xu et al., 2009; Gupta et al., 2015), to obtain a common set of sources, ordered in the same way among different subjects, a group ICA (considering all PPA patients) was calculated using the GIFT toolbox (Group ICA toolbox v4.0c; https://trendscenter.org/software/gift/) (Calhoun et al., 2001; Iraji et al., 2021), with neural network algorithm (Infomax) that attempts to minimize the mutual information of the network outputs (McKeown et al., 1998). The component number was estimated to be 12, based on the minimum description length principle (Li et al., 2007) and the statistical reliability of the sources decomposition was tested using the ICASSO toolbox (Himberg et al., 2004) by running Infomax 10 times with different initial conditions and bootstrapped data sets. Individual source maps were converted to *Z*-scores before entering group statistics, to obtain voxel values comparable across subjects. As previously described for SBM (Xu et al., 2009), the mixing matrix (containing the loading parameters for each subject and each source) was used for statistical analyses. The source matrix was used for visualization, by scaling each map to unit standard deviation (SBM Z-map) and thresholding at |Z| >2.0. The maps of significant sources were then superimposed onto the MNI-normalized template brain. Partial correlation analyses testing the correlation between individual scores for each language test and individual loading parameters for each source were performed. Age, gender, educational level (years of schooling), total GMV and FTLD-modified CDR (sum of boxes) were considered nuisance variables. Taking into account the potential statistical relationship among (i) the considered variables (i.e., verbal fluency tasks, AAT naming subtest, and SAND subtests), (ii) the number of source maps and (iii) the number of statistical tests performed, a statistical threshold corrected for multiple comparisons was implemented [*p* < 0.05, family-wise-error (FWE) correction (number of sources ^*^ number of tests)] (Bonferroni-Holms methods) (Nichols and Hayasaka, 2003).

### Statistical analysis

Continuous and categorical variables are reported as mean (± standard deviation) and n (%), respectively. Statistical analyses were performed using SPSS (v.24; SPSS, IBM).

## Results

### Participant characteristics

In the present study, we considered 31 patients fulfilling the current diagnostic criteria for imaging-supported avPPA (left posterior fronto-insular atrophy or hypometabolism) (mean age = 68.2 ± 8.2, female = 64.5%). Demographical, clinical, neuropsychological and language characteristics of the avPPA group are reported in Tables 1, 2.

**Table 2:** Clinical, neuropsychological, and language features of the sample.

	**Raw score**	**Range**	**Adjusted score**	**Cut-off (in the normal range if)**
	**Mean (SD)**	**Min-Max**	**Mean (SD)**	
**Clinical assessment**				
Cognitive reserve index questionnaire (CRI-q)				
CRI-total score	107.5 (19.6)	-	-	-
CRI-education	101.0 (12.7)	-	-	-
CRI-working activity	101.5 (18.5)	-	-	-
CRI-leisure time	114.8 (23.9)	-	-	-
Frontotemporal Dementia—clinical dementia rating score (FTLD-modified CDR, sum of boxes)	5.9 (4.4)	0 (excellent)-24 (poor)	-	-
Frontal behavioral inventory (FBI)	14.9 (10.3)	0 (excellent)-72 (poor)	-	-
Beck depression inventory (BDI-II)	8.7 (4.8)	0 (excellent)-63 (poor)	-	-
Stroke and Aphasia quality of life scale (SAQOL-39)				
Physical	4.6 (0.4)	1 (poor)-5 (excellent)	-	-
Psychosocial/mood	3.8 (0.8)	1 (poor)-5 (excellent)	-	-
Communication	3.2 (0.7)	1 (poor)-5 (excellent)	-	-
Energy	3.8 (1.0)	1 (poor)-5 (excellent)	-	-
Total score	4.1 (0.4)	1 (poor)-5 (excellent)	-	-
Speech questionnaire				
Production	9.5 (3.5)	0 (poor)-14 (excellent)	-	-
Comprehension	3.7 (1.5)	0 (poor)-5 (excellent)	-	-
Communication assessment scale	1.8 (1.3)	0 (poor)-5 (excellent)	-	-
**Screening for dementia**				
Mini Mental State Examination (MMSE)	**17.9 (6.6)**	0 (poor)-30 (excellent)	**16.5 (6.7)**	> 23
**Memory**
Story recall	**2.9 (2.8)**	0 (poor)-28 (excellent)	**3.5 (3.3)**	> 7.5
Rey-Osterrieth complex figure—recall	**4.3 (4.1)**	0 (poor)-36 (excellent)	**7.9 (5.1)**	> 9.46
**Language**				
Verbal Fluency, phonemic	**9.0 (8.0)**	-	**10.9 (9.5)**	> 16
Verbal Fluency, semantic	**10.9 (7.8)**	-	**14.3 (9.3)**	> 24
Aachener Aphasie test (AAT)—naming subtest	**80.4 (24.4)**	0 (poor)-120 (excellent)	-	> 103
Object naming task IPNP (accuracy, %)	47.5 (26.5)	-	-	-
Action naming task IPNP (accuracy, %)	42.8 (26.5)	-	-	-
**Screening for aphasia in NeuroDegeneration**				
Picture naming				
Living	3.968 (2.6)	0 (poor)-7 (excellent)	4.078 (2.6)	> 3.829
Non-living	**3.726 (2.6)**	0 (poor)-7 (excellent)	-	> 5
Total score	**7.694 (5.0)**	0 (poor)-14 (excellent)	**7.835 (5.0)**	> 9.969
Auditory sentence comprehension				
Total score	**5.323 (2.2)**	0 (poor)-8 (excellent)	**5.336 (2.2)**	> 6.157
Single-word comprehension				
Living	5.393 (1.0)	0 (poor)-6 (excellent)	5.415 (1.0)	> 5.048
Non-living	**4.821 (1.2)**	0 (poor)-6 (excellent)	**4.819 (1.2)**	> 4.876
Total score	**10.226 (2.0)**	0 (poor)-12 (excellent)	**10.239 (1.9)**	> 10.258
Words and Non-words repetition				
Words	5.179 (1.4)	0 (poor)-6 (excellent)	5.192 (1.4)	> 4.928
Non-words	1.179 (1.3)	0 (poor)-4 (excellent)	1.276 (1.3)	> 0.483
Total score	6.452 (2.3)	0 (poor)-10 (excellent)	6.629 (2.3)	> 6.349
Sentence repetition				
Predictable	**0.893 (0.7)**	0 (poor)-3 (excellent)	**0.927 (0.8)**	> 1.001
Unpredictable	**0.536 (0.7)**	0 (poor)-3 (excellent)	**0.544 (0.7)**	> 0.784
Total score	**1.516 (1.3)**	0 (poor)-6 (excellent)	**1.558 (1.3)**	> 2.455
Reading				
Words	10.538 (2.2)	0 (poor)-12 (excellent)	10.552 (2.2)	> 10.106
Non-words	2.808 (1.0)	0 (poor)-4 (excellent)	2.829 (1.0)	> 2.228
Total score	**13.346 (2.7)**	0 (poor)-16 (excellent)	**13.404 (2.8)**	> 13.489
Writing				
Information Units (IUs)	2.941 (1.6)	0 (poor)-6 (excellent)	3.121 (1.6)	> 2.132
Semantic association				
Total score	2.429 (0.9)	0 (poor)-4 (excellent)	2.448 (1.0)	> 1.166
Picture description				
Information Units (IUs)	3.769 (1.8)	0 (poor)-8 (excellent)	-	> 3
Visuo-constructional abilities				
Rey-Osterrieth complex figure—copy	**15.8 (10.7)**	0 (poor)-36 (excellent)	**17.2 (10.2)**	> 28.87
Attentional and Executive Functions
Trail Making Test—part A (sec)	**174.0 (106.8)**	-	**155.5 (101.6)**	< 127
Trail Making Test—part B (sec)	**495.1 (167.1)**	-	**443.0 (163.0)**	< 294

Mean raw scores and adjusted scores are reported. If the test provided the correction grid, the raw score is adjusted to remove the influence of age and/or education, and/or sex. -: correction grid is not available. Standard Deviation (SD) between brackets. Cut-off scores according to Italian normative data are reported. The bold font indicates pathological scores.

IPNP, international picture naming project; Min, minimum; Max, maximum.

Regarding language assessment, almost all the avPPA patients exhibited difficulties in phonemic (77%) and semantic (84%) verbal fluency tasks and in the Aachener Aphasie Test (AAT)—naming subtest (87%). Moreover, with respect to SAND battery, more than half of the avPPA patients showed an impairment in picture naming non-living score (65%), auditory sentence comprehension (65%) and sentence repetition subtests (unpredictable = 55%; total score = 77%).

Otherwise, the majority of the patients performed within the normal range on picture naming subtests (total score = 52%; living score = 61%), single-word comprehension subtests (living score = 74%; non-living score = 61%; total score = 58%), words and non-words repetition subtests (words = 81%; non-words = 65%; total score = 58%), sentence repetition subtest (predictable = 52%); reading subtests (words = 69%; non-words = 72%; total score = 62%), writing-Information Units (70%), semantic association (87%) and picture description-Information Units (55%). Interestingly, the qualitative error analysis of language performance in the SAND battery is in line with the current diagnostic criteria of avPPA (Gorno-Tempini et al., 2011), confirming in these patients agrammatism, inconsistent speech sound errors and apraxia of speech. To evaluate the presence of agrammatism, errors of syntactic nature were considered, showing that 84% of patients made morphological errors in the auditory sentence comprehension test, 77% of patients showed grammatical/syntactic errors in the writing test, 45% of subjects produced grammatical/syntactic errors in the picture description test, while 16% of patients made morphological errors in sentence repetition test. Regarding the speech sound errors, the qualitative error analysis revealed that most subjects made phonological errors rather than phonetic ones. Specifically, the presence of phonological errors (i.e., deletions, substitutions, insertions, and transpositions) was reported in the repetition subtests (non-words = 90%; words = 35%), reading subtests (non-words = 71%; words = 45%), picture naming test (48%), sentence repetition test (39%) and picture description test (35%). Moreover, distortions (phonetic errors) were observed in 29% of patients during the picture description test, 16% of subjects during the sentence repetition test and 13% of patients during the picture naming test. Distortions were not found in the other oral production tasks (reading and words and non-words repetition subtests).

### Univariate VBM-SPM analysis

**Figure 1:**
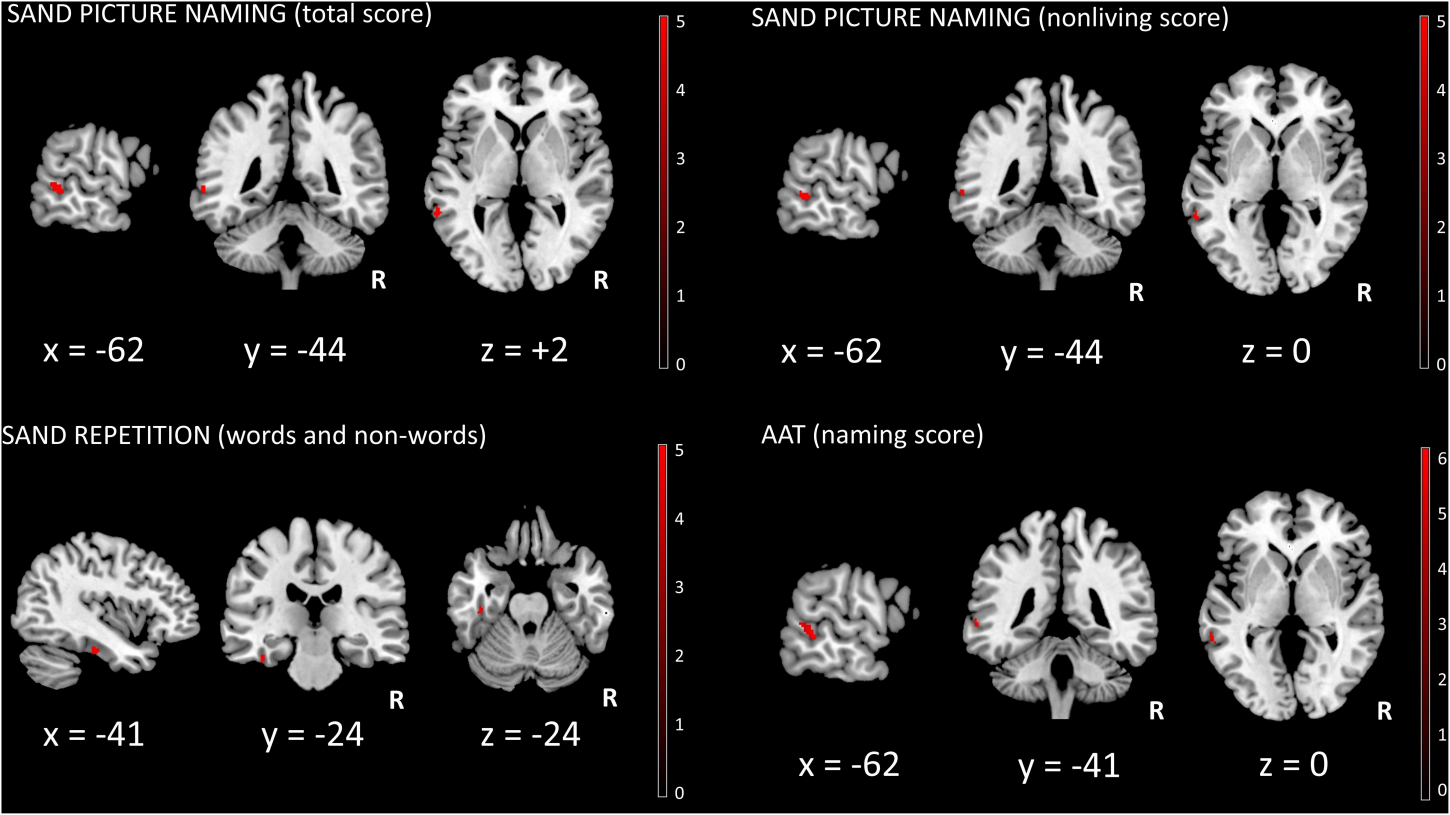
SPM univariate analysis. Significant clusters (surviving FWE whole-brain correction, *p* < 0.05) were superimposed on a standardized T1 MRI template (MRIcron, https://www.nitrc.org/projects/mricron). R, right; SAND, Screening for Aphasia in NeuroDegeneration battery; AAT, Aachener Aphasie Test.

At the pre-established threshold, a significant direct correlation between picture naming (total score) and left middle temporal gyrus [(*x, y, z*: −62, −44, +2) *T* = 5.60, 48 voxels, *p* = 0.02 FWE whole-brain] was evident (Figure 1). For picture naming (non-living score), a significant direct correlation with left middle temporal gyrus [(*x, y, z*: −62, −44, 0) *T* = 5.42, 27 voxels, *p* = 0.03 FWE whole-brain] (Figure 1) was also demonstrated. No significant correlation for picture naming (living score) was demonstrated. For words and non-words repetition (total score), a significant direct correlation with left fusiform gyrus [(*x, y, z*: −41, −24, −24) *T* = 5.67, 19 voxels, *p* = 0.03 FWE whole-brain] was present (Figure 1). For sentence repetition (total score), auditory sentence comprehension, and single-word comprehension (total score), no significant correlations were evident. Moreover, no significant correlations between sources and fluencies (phonemic and/or semantic) were demonstrated, whereas a significant direct correlation between AAT (naming subtest) and left middle temporal gyrus was shown [(*x, y, z*: −62, −39, −2) *T* = 6.17, 78 voxels, *p* = 0.009 FWE whole-brain] (Figure 1). Finally, no significant correlations between sources and disease duration as well as FTLD-modified CDR scores were evident.

### Multivariate SBM analysis

**Figure 2:**
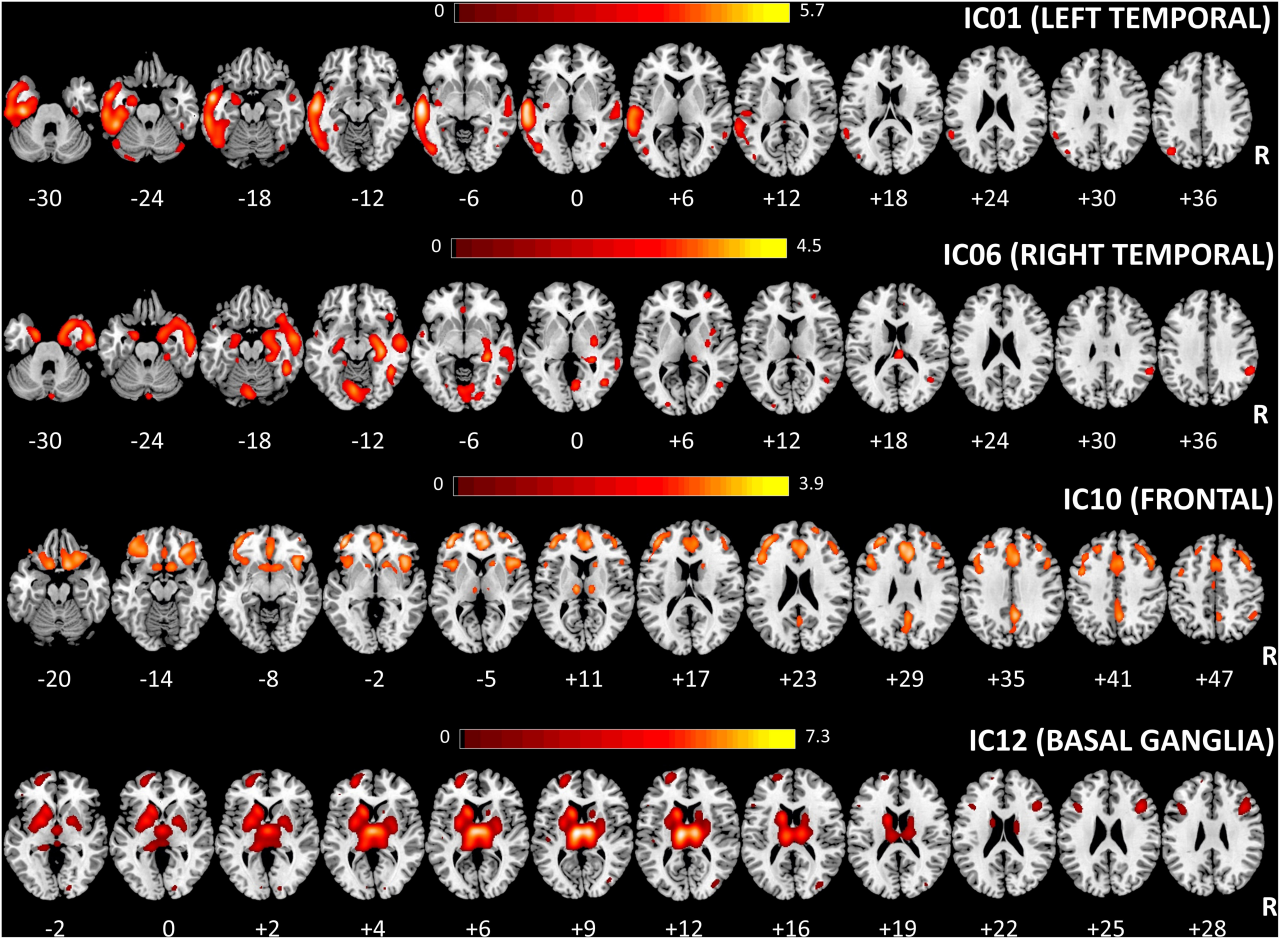
Spatial maps of the four considered sources for multivariate SBM analysis are shown, superimposed on an axial T1 MRI standard template (MRIcron, https://www.nitrc.org/projects/mricron). The number under each slice represented the standardized z coordinate. Voxels above the threshold of |Z| > 2.0 are shown. R, right; SBM, source-based morphometry.

Four out of the twelve independent components were considered as sources of interest after excluding four artifact components (i.e., signal near the external boundary of the brain or appearing primarily in ventricles or white matter areas) and four other sources not related to fronto-temporal regions [visual cortex (2) and cerebellar (2)] (see Supplementary Figure 1). The four sources of interest include temporal [left (IC01) and right (IC06)], frontal (IC10), and basal ganglia (IC12) regions (Figure 2). The left temporal source (IC01) also encompassed small clusters located in the right temporal cortex, whereas the frontal source (IC10) included several regions (anterior cingulate, insula bilaterally) belonging to salience network configuration (see Supplementary Tables 1–4 for the anatomical description of the four sources and Figure 2 for representation).

These four sources were considered for correlation analysis (using individual loading parameters) with those language tests that were specifically altered in avPPA. In particular, a significant direct correlation between IC01 (left temporal) and picture naming subtests (total score, living score, and non-living score), words and non-words repetition (total score) and AAT (naming subtest score) were evident (see Table 3 for details) after multiple comparisons correction (FWE 0.05). No significant correlations between sources and fluency tests (phonemic and/or semantic) were identified. A significant inverse correlation between FTLD-modified CDR scores and frontal (*r* = −0.421, *p* = 0.029) as well as basal ganglia (*r* = −0.517, *p* = 0.006) sources were also shown (not corrected for multiple comparisons).

**Table 3:** Partial correlation analysis in avPPA between language tests and SBM sources.

	**IC01 left temporal**	**IC06 right temporal**	**IC10 frontal**	**IC12 Basal Ganglia**
SAND picture naming, total	0.0002 [0.01] (+0.666)	0.06	0.85	0.13
SAND picture naming, living	0.0003 [0.02] (+0.651)	0.037 (+0.411)	0.685	0.166
SAND picture naming, non-living	0.001 [0.04] (+0.627)	0.122	0.98	0.13
SAND auditory sentence comprehension	0.075	0.343	0.613	0.956
SAND single-word comprehension, total	0.028 (+0.430)	0.444	0.263	0.961
SAND words/non-words repetition, total	0.0004 [0.02] (+0.671)	0.06	0.826	0.03 (-0.427)
SAND sentence repetition, total	0.012 (0.486)	0.308	0.582	0.078
Verbal Fluency, phonemic	0.817	0.947	0.141	0.956
Verbal Fluency, semantic	0.016 (+0.469)	0.207	0.800	0.129
AAT naming, total	0.0004 [0.02] (+0.644)	0.289	0.435	0.175

SAND, screening for Aphasia in NeuroDegeneration battery; AAT, Aachener Aphasie test; IC, independent component(sources) from SBM analysis; SBM, source-based morphometry; uncorrected (for multiple comparisons) p is reported for each comparison; corrected p (for multiple comparisons) is reported between squared brackets; when significant (p < 0.05), Pearson's correlation (r) value was reported between round brackets. Statistical comparisons surviving multiple comparisons corrections (p < 0.05 Holm-Bonferroni) were highlighted in light gray.

## Discussion

The aim of the present study was to evaluate the correlation between brain structure and linguistic scores in a group of avPPA patients through an univariate/multivariate approach. The enrolled avPPA patients exhibited difficulties in verbal fluency tasks, picture naming (AAT and SAND subtests), and auditory sentence comprehension and sentence repetition subtests from the SAND battery. Interestingly, the qualitative error analysis of language performance in the SAND battery is in line with the current diagnostic criteria of avPPA (Gorno-Tempini et al., 2011), confirming in these patients agrammatism, inconsistent speech sound errors and apraxia of speech.

In the evaluation of PPAs, different components of language need to be assessed to obtain a precise clinical diagnosis. Since PPA refers to heterogeneous neurodegenerative disorders, characterized by specific deteriorations of language (Rohrer et al., 2008), sensitive instruments for language evaluation in these patients are needed to improve the diagnostic pathway and to select the better treatment for each PPA patient. In particular, avPPA is characterized by effortful speech and agrammatic language production together with sentence comprehension difficulties, svPPA is marked by impaired semantic knowledge, whereas lvPPA is distinguished by slowed speech output, word-finding pauses and phonemic paraphasias. Beyond the clinical application, reliable screening tests that catch key language features of PPA (i.e., SAND) can represent an interesting tool to better elucidate how the language system works in the different clinical variants of PPA.

In the present study, we have taken advantage of a combined univariate/multivariate neuroimaging approach (univariate SPM-VBM/multivariate SBM) to explore the brain structural underpinnings of the language impairment that is characterized by a cohort of avPPA patients. First, in a fully data-driven fashion, the SBM approach has been able to separate the multivariate signal of whole-brain GMV into maximally independent sources that partially resemble brain large-scale networks derived from literature data, in particular for the temporal network. Indeed, our findings demonstrated that specific language features captured by SAND (picture naming subtests: total score, living score, non-living score and words-non words repetition subtest: total score) and AAT battery (naming subtest-total score) were positively correlated with GMV in a series of left-sided cortical regions (fusiform gyrus, parahippocampal gyrus, inferior and medial temporal gyri and inferior and superior parietal lobuli) that are globally included in this left temporal network. Interestingly, a trend (not surviving multiple comparisons correction) toward a significant positive correlation with the left temporal network was also evident for the remaining language tests (auditory sentence comprehension, single-word comprehension, and sentence repetition-total score), potentially suggesting the pivotal role of the left temporal network in sustaining language features that are impaired in avPPA. Moreover, this left temporal network also encompassed small contralateral clusters in the right hemisphere, highlighting the structural and functional interplays of temporal regions in language functioning (Brambati et al., 2006; Breining et al., 2022).

Besides this multivariate analysis, classical univariate analysis (SPM-VBM) revealed that different regions were linked with different language tests (naming total score of SAND and AAT and SAND living score: left middle temporal gyrus; words and non-words repetition-total score: left fusiform gyrus). For naming, previous literature data (Brambati et al., 2006; Migliaccio et al., 2016; Bruffaerts et al., 2020; Breining et al., 2022) on different neurodegenerative diseases demonstrated a specific involvement of bilateral temporal regions (considering total scores), with a differential involvement for living (right parahippocampal region) and non-living (left middle temporal gyrus) target words. This is partially in line with our findings (in particular for total scores from the naming subtest of SAND and AAT) with contrasting results for living/non-living categories. However, different language screening tools were used as well as a mixed group of neurodegenerative patients and typical controls. Our finding matches the fact that left temporal regions seem to respond preferentially to tools (Damasio et al., 1996; Cappa et al., 1998; Okada et al., 2000; Joseph, 2001; Giussani et al., 2011). Further studies with multimodal approaches might shed light on this interesting anatomical specificity. From this point of view, this is the first study that assessed structural neuroimaging correlates of naming specifically in avPPA, potentially demonstrating a left-sided dominance (in particular for temporal regions) for naming functioning. On the other hand, sentence repetition (total score) has been linked to the left temporoparietal junction (Amici et al., 2007; Lukic et al., 2019; Miller et al., 2021) with also the involvement of the middle and anterior temporal gyri (Fedorenko et al., 2010; Friederici and Gierhan, 2013). Furthermore, the semantic fluency test demonstrated a slight correlation with the left temporal network, not surviving multiple comparisons. This is partially in line with previous literature data (Riello et al., 2021) that described neural substrates of semantic fluency in the left temporal gyrus. Overall, our results corroborate the specificity of language tests like SAND and AAT in demonstrating a higher magnitude of correlation with the left temporal network. Moreover, although the AAT battery was developed to examine the language performance of stroke aphasic patients (Biniek et al., 1992), the results of our study revealed that it is a valid instrument for detecting impairment in PPA as well. However, the picture naming task includes in the SAND battery provides a distinction of items into living and non-living, offering the possibility to investigate a possible category effect in these patients. Thus, univariate and multivariate approaches supported the pivotal role of a left-sided cortical network in sustaining specific language features directly related to avPPA. This approach (multivariate analysis for the definition of structural covariance networks and univariate analysis to highlight peak clusters of correlations) applied to a more homogeneous clinical sample can increase our knowledge of the neural basis of language functioning in neurodegenerative diseases. We acknowledge that our study has some limitations. First, given that the number of patients included in this pilot study was relatively small, the findings reported here need to be reproduced in larger cohorts before drawing firm conclusions, also to confirm those findings excluded by the multiple comparison correction. A larger population would be necessary to confirm the present results also considering the limited number of items included in the screening language battery applied. Moreover, the utilization of structural covariance (through the SBM approach) only partially disentangles the role of the left temporal network and was not able to identify a specific frontal component focused on the left inferior frontal gyrus (as the core region damaged in avPPA), as well as the relationship among these regions, usually involved in language performances in PPA (Gunawardena et al., 2010). From this point of view, dynamic functional connectivity for the study of time-varying spatial patterns of brain networks might further elucidate the neuroanatomical mechanisms sustaining specific language features in avPPA (Iraji et al., 2019; Premi et al., 2020) as well as the “dynamic” role of anatomical hubs for avPPA (pars triangularis and pars opercularis of the left inferior frontal gyrus, left superior temporal gyrus) (Wilson et al., 2010). Furthermore, the lack of a correlation between frontal regions and language scores may reflect the unavailability of objective and quantifiable measures capable of capturing the typical articulatory features of the avPPA variant in the selected language assessment.

We decided to conduct the present study recruiting only avPPA patients to investigate a quite homogenous sample, but future studies should investigate the correlation between linguistic deficits, as assessed by SAND, and structural imaging also in svPPA and lvPPA to deeply understand the neural bases of the specific language difficulties of each PPA variant. Finally, the linguistic abilities of PPA patients of each variant at different stages of the disease would be interesting to be investigated to assess the effect of neurodegeneration progression on specific language difficulties.

In conclusion, the present findings might potentially expand our knowledge of the neuroanatomical basis of this neurodegenerative condition.

## Data availability statement

The datasets presented in this study can be found in online repositories. The names of the repository/repositories and accession number(s) can be found below: doi: 10.5281/zenodo.6874925.

## Ethics statement

The studies involving human participants were reviewed and approved by Ethics Committees of the Istituto di Ricovero e Cura a Carattere Scientifico (IRCCS) Centro San Giovanni di Dio, Fatebenefratelli, Brescia, Italy and of ASST Spedali Civili, Brescia. The patients/participants provided their written informed consent to participate in this study.

## Author contributions

EP, MC, EG, IP, GB, YG, IL, IM, MP, AI, VC, AA, BB, and RM: conception and methodology and writing—review and editing. EP, MC, EG, IP, BB, and RM: data curation. EP, MC, and RM: writing—original draft preparation. All authors contributed to the article and approved the submitted version.

## Funding

This study was supported by the Italian Ministry of Health (GR-2018-12365105) and the National Institutes of Health (RF1AG063153).

## Conflict of interest

The authors declare that the research was conducted in the absence of any commercial or financial relationships that could be construed as a potential conflict of interest.

## Publisher's note

All claims expressed in this article are solely those of the authors and do not necessarily represent those of their affiliated organizations, or those of the publisher, the editors and the reviewers. Any product that may be evaluated in this article, or claim that may be made by its manufacturer, is not guaranteed or endorsed by the publisher.
