# Supplementary material for: Neuroanatomical correlates of screening for aphasia in NeuroDegeneration (SAND) battery in non-fluent/agrammatic variant of primary progressive aphasia
Source: Front Aging Neurosci. 2022 Oct 31;14:942095. doi: 10.3389/fnagi.2022.942095 (PMC9660243; doi:10.3389/fnagi.2022.942095)

**Supplementary Table 1.** IC01 (left temporal) derived from SBM analysis.

| **BA** | **Region** | **MNI** | **Volume (cc)** |
| --- | --- | --- | --- |
| 18, 19, 20, 36, 37 | Fusiform Gyrus | 5.6 (-37, -20, -24)/2.9 (40, -69, -13) | 8.0/1.2 |
| 19, 28, 34, 35, 36, 37 | Parahippocampal Gyrus | 5.3 (-37, -23, -22)/2.8 (30, -44, -4) | 6.8/2.0 |
| 19, 20, 21, 37 | Inferior Temporal Gyrus | 5.4 (-42, 0, -35)/2.3 (45, -45, -18) | 6.7/0.3 |
| 7, 19, 39 | Precuneus | 3.5 (-24, -60, 49)/2.1 (1, -62, 39) | 3.5/0.3 |
| 20, 21, 22, 37, 38, 39 | Middle Temporal Gyrus | 6.2 (-58, -15, -6)/3.1 (56, -3, -10) | 20.4/4.7 |
| 7 | Superior Parietal Lobule | 4.0 (-25, -63, 46)/2.6 (36, -68, 45) | 2.8/0.2 |
| 20, 28, 34, 36, 38 | Uncus | 5.1 (-37, -17, -27)/2.4 (30, -13, -27) | 2.6/0.4 |
| 19, 37 | Middle Occipital Gyrus | 3.8 (-50, -63, -8)/2.9 (43, -64, 3) | 2.4/0.6 |
| 13, 21, 22, 38, 39, 41, 42 | Superior Temporal Gyrus | 5.9 (-59, -22, -1)/3.3 (59, -22, -1) | 17.6/3.5 |
| 7, 39, 40 | Inferior Parietal Lobule | 3.2 (-34, -61, 40)/2.4 (39, -66, 42) | 1.7/0.3 |
| 40 | Supramarginal Gyrus | 3.2 (-58, -44, 26)/0 | 1.1/0.0 |
| 13 | Insula | 2.7 (-37, -9, -5)/0 | 1.1/0.0 |
| 41, 42 | Transverse Temporal Gyrus | 3.1 (-58, -20, 11)/0 | 0.8/0.0 |
| 39 | Angular Gyrus | 2.9 (-43, -65, 34)/0 | 0.6/0.0 |
| 13, 47 | Inferior Frontal Gyrus | 2.6 (-40, 15, -18)/0 | 0.5/0.0 |

BA: Broadmann area; L: left; R: right; MNI: Montreal Neurological Institute coordinates system: the value of the random effect was reported as well as the location (expressed in MNI coordinates) of the peak for each side (L/R). cc: cluster dimension expressed in cubic centimeters.

**Supplementary Table 2.** IC06 (right temporal) derived from SBM analysis.

| **BA** | **Region** | **MNI** | **Volume (cc)** |
| --- | --- | --- | --- |
| 20, 28, 34, 36, 38 | Uncus | 5.2 (-22, -2, -33)/5.5 (24, -2, -34) | 2.8/3.6 |
| 18 | Lingual Gyrus | 3.9 (-7, -76, -6)/3.4 (1, -79, -6) | 0.9/0.4 |
| 20, 21, 37 | Inferior Temporal Gyrus | 3.7 (-31, -8, -35)/5.7 (31, -8, -35) | 0.3/2.7 |
| 34, 35, 36 | Parahippocampal Gyrus | 3.2 (-22, -9, -12)/4.2 (28, -15, -11) | 0.3/1.9 |
| 20, 21, 38 | Middle Temporal Gyrus | 0/4.6 (36, 0, -39) | 0.0/4.9 |
| 38 | Superior Temporal Gyrus | 0)/5.0 (25, 7, -34) | 0.0/3.3 |
| 20, 37 | Fusiform Gyrus | 0/4.8 (55, -7, -26) | 0.0/1.2 |
| 47 | Inferior Frontal Gyrus | 0/3.3 (43, 23, -12) | 0.0/0.2 |

BA: Broadmann area; L: left; R: right; MNI: Montreal Neurological Institute coordinates system: the value of the random effect was reported as well as the location (expressed in MNI coordinates) of the peak for each side (L/R). cc: cluster dimension expressed in cubic centimeters.

**Supplementary Table 3.** IC10 (frontal) derived from SBM analysis.

| **BA** | **Region** | **MNI** | **Volume (cc)** |
| --- | --- | --- | --- |
| 8, 9, 10, 11, 46 | Middle Frontal Gyrus | 4.0 (-42, 45, -1)/3.6 (30, 34, -13) | 3.4/1.2 |
| 9, 10, 25, 32 | Medial Frontal Gyrus | 4.1 (-3, 53, 2)/3.7 (12, 15, -17) | 1.3/1.2 |
| 10, 24, 32 | Anterior Cingulate | 3.9 (-3, 49, -1)/3.8 (6, 44, 3) | 1.1/2.3 |
| 10, 45, 46, 47 | Inferior Frontal Gyrus | 3.5 (-43, 42, 2)/3.7 (27, 31, -14) | 0.9/2.2 |
| 31, 32 | Cingulate Gyrus | 3.7 (-3, 35, 26)/3.7 (4, 26, 28) | 0.4/1.4 |
| 13 | Insula | 3.4 (-39, 16, 2)/3.6 (42, 18, 1) | 0.4/0.5 |
| 7, 31 | Precuneus | 3.1 (0, -48, 31)/3.7 (4, -48, 34) | 0.1/0.3 |
| 11 | Rectal Gyrus | 3.1 (-10, 18, -19)/3.1 (9, 22, -19) | 0.1/0.2 |
| 9 | Superior Frontal Gyrus | 3.1 (-34, 47, 17)/3.1 (33, 35, 33) | 0.1/0.1 |
| 9 | Precentral Gyrus | 0/3.3 (43, 21, 36) | 0.0/0.2 |

BA: Broadmann area; L: left; R: right; MNI: Montreal Neurological Institute coordinates system: the value of the random effect was reported as well as the location (expressed in MNI coordinates) of the peak for each side (L/R). cc: cluster dimension expressed in cubic centimeters.

**Supplementary Table 4.** IC12 (frontal) derived from SBM analysis.

| **BA** | **Region** | **MNI** | **Volume (cc)** |
| --- | --- | --- | --- |
| **.** | Thalamus | 8.5 (-7, -14, 10)/8.0 (9, -11, 9) | 5.3/4.5 |
| **.** | Lentiform Nucleus | 4.7 (-24, 2, 3)/3.7 (19, 3, 0) | 3.5/1.2 |
| **.** | Caudate | 5.9 (-13, 13, 8)/3.3 (15, -12, 19) | 3.1/0.2 |
| 28, 34 | Parahippocampal Gyrus | 4.2 (-24, -14, -13)/0 | 1.7/0.0 |
| 10 | Superior Frontal Gyrus | 4.2 (-25, 54, 3)/0 | 0.9/0.0 |
| **.** | Middle Frontal Gyrus | 3.3 (-25, 51, 6)/4.0 (42, 22, 24) | 0.2/0.4 |

BA: Broadmann area; L: left; R: right; MNI: Montreal Neurological Institute coordinates system: the value of the random effect was reported as well as the location (expressed in MNI coordinates) of the peak for each side (L/R). cc: cluster dimension expressed in cubic centimeters.

**Supplementary Figure 1.** Spatial maps of the remaining sources (not considered for the statistical analysis) of SBM analysis are shown, superimposed on an axial T1 MRI standard template (MRIcron, https://www.nitrc.org/projects/mricron). Green components: artifacts; light blue components: cerebellum; yellow components: visual network. Voxels above the threshold of │Z│>2.0 are shown. SBM: source-based morphometry.


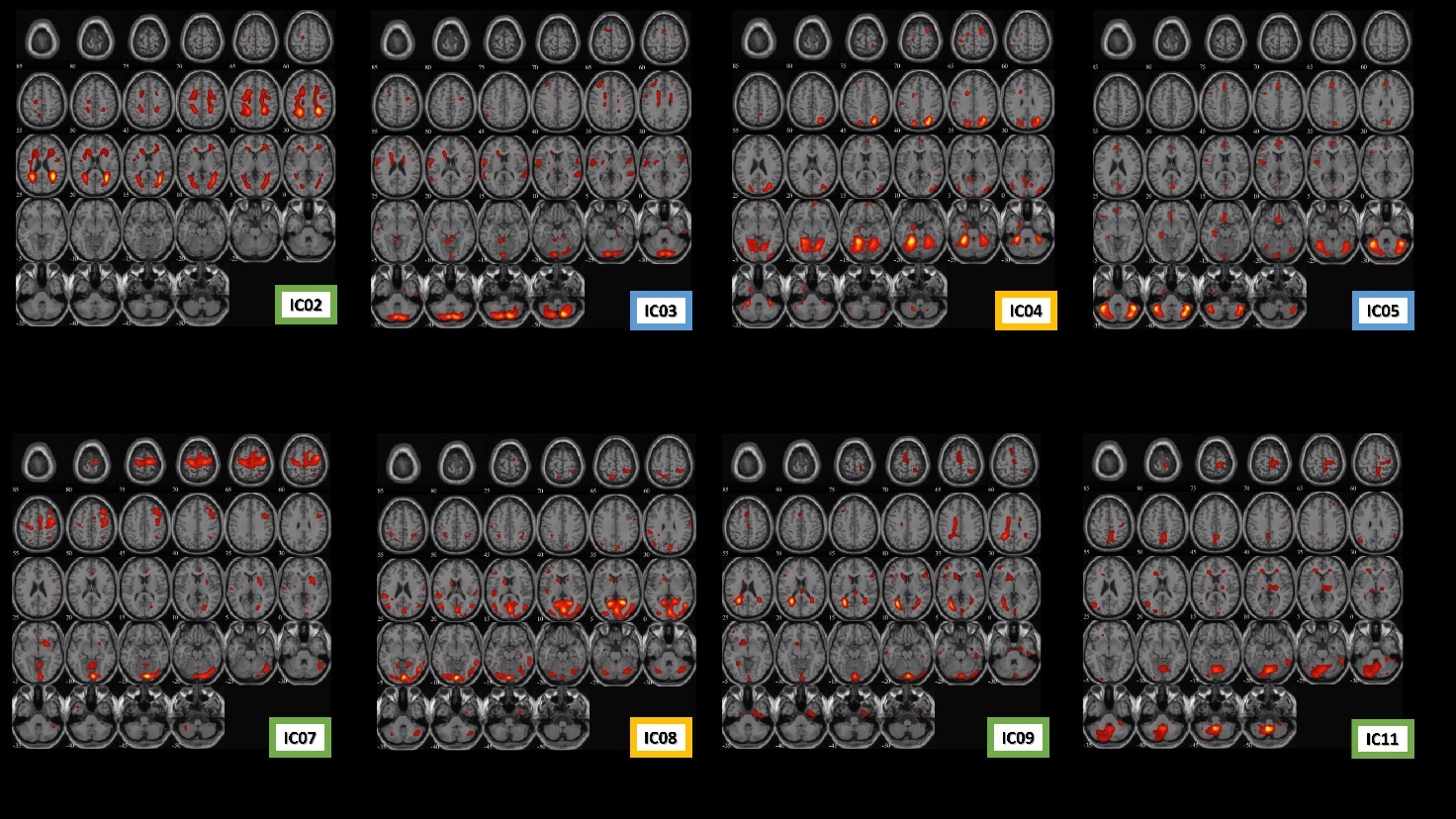

Supplement: Supplementary file 1 [file Data_Sheet_1.docx]
